# Supplementary material for: Identification of Freezing-Responsive microRNAs and Their Targets in Chinese Jujube by Small RNA and Degradome Sequencing
Source: Int J Mol Sci. 2026 Sep 4;27(17):7912. doi: 10.3390/ijms27177912 (PMC13566890; doi:10.3390/ijms27177912)
Supplement: Supplementary file 1 [file ijms-27-07912-s001.zip › Supplementary Material S2/novel_miR_56.pdf]

Provisional ID	:	novel_miR_56
Score total	:	1427.4
Score for star read(s)	:	3.9
Score for read counts	:	1425.6
Score for mfe	:	0.1
Score for randfold	:	-2.2
Score for cons. seed	:	
Total read count	:	2808
Mature read count	:	2796
Loop read count	:	0
Star read count	:	12

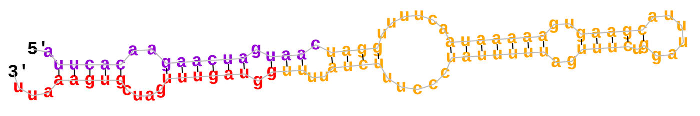

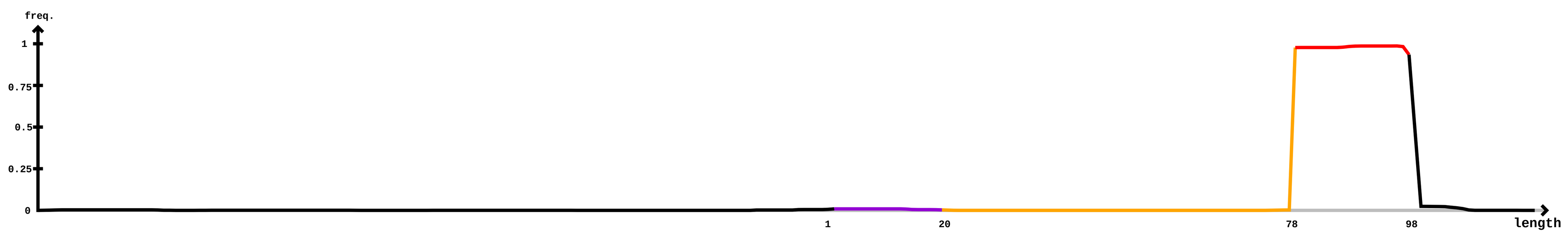

Star Mature

```
5'- guaaaguagcuccugggugggaaacuacuccuuugauggguaucgcaauggcccuuuuugcauuauuccuaucuauuauuuuggagauuuauacuucuuccauuuuacuggacaaauuuggaaugaauuagauucacaagaacuaguaacuagguuuucaauaaaaagugaagcauuuaggucuuugauuuuuaucccuuucuauuuugguaguuugaucgugaaauuucuuuguuuuuguaguuuugaa -3'  obs
    guaaaguagcuccugggugggaaacuacuccuuugauggguaucgcaauggcccuuuuugcauuauuccuaucuauuauuuuggagauuuauacuucuuccauuuuacuggacaaauuuggaaugaauuagauucacaagaacuaguaacuagguuuucaauaaaaagugaagcauuuaggucuuugauuuuuaucccuuucuauuuugguaguuugaucgugaaauuucuuuguuuuuguaguuuugaa       exp
    ...((((.(((((((((((((((..(((.((......)))))..((((.........))))....))))))))))......))))....)))))..((((.....))))((((.(((.((.((...((((((((..((((((.(((.((((......(((((((..(((((......).))))..)))))))).....))))..))).))))))....)))))...)))...)).)).))).))))..   reads   mm   sample
    guaaaguagcuccugggu................................................................................................................................................................................................................................................   2   0   LT-3
    guaaaguagcuccugggug...............................................................................................................................................................................................................................................   1   0   JCK-1
    guaaaguagcuccugggug...............................................................................................................................................................................................................................................   1   0   JCK-2
    .uaaaguagcuccugggug...............................................................................................................................................................................................................................................   3   0   JCK-2
    ..aaaguagcuccuggguggg.............................................................................................................................................................................................................................................   2   0   LT-1
    .........................uacuccuuugauggguaucgcaauggc......................................................................................................................................................................................................   1   0   LT-2
    ...........................cuccuuugauggguaucgcaaugg.......................................................................................................................................................................................................   1   0   JT-3
    ................................................................auuccuaucuauuauuuuggagau..................................................................................................................................................................   1   0   JCK-2
    ......................................................................................................................uggaaugaauuagauucacaagaacu..........................................................................................................   1   0   LT-2
    ......................................................................................................................uggaaugaauuagauucacaagaacu..........................................................................................................   2   0   LT-3
    ......................................................................................................................uggaaugaauuagauucacaagaacua.........................................................................................................   2   0   LT-2
    ......................................................................................................................uggaaugaauuagauucacaagaacuagua......................................................................................................   1   0   LT-1
    .............................................................................................................................aauuagauucacaagaac...........................................................................................................   3   0   LCK-3
    .............................................................................................................................aauuagauucacaagaacu..........................................................................................................   1   0   JCK-2
    .............................................................................................................................aauuagauucacaagaacu..........................................................................................................   1   0   JCK-3
    .............................................................................................................................aaNuagauucacaagaacu..........................................................................................................   1   1   LT-2
    .............................................................................................................................aauuagauucacaagaacu..........................................................................................................   1   0   LT-2
    ..............................................................................................................................auuagauucacaagaacu..........................................................................................................   1   0   JCK-2
    ..................................................................................................................................gauucacaagaacuaguaa.....................................................................................................   2   0   JT-2
    ..................................................................................................................................gauucacaagaacuaguaa.....................................................................................................   2   0   JT-3
    ...................................................................................................................................auucacaagaacuaguaac....................................................................................................   4   0   JCK-1
    ...................................................................................................................................auucacaagaacuaguaac....................................................................................................   1   0   LT-3
    ...................................................................................................................................auucacaagaacuaguaacu...................................................................................................   2   0   LT-3
    ...................................................................................................................................auucacaagaacuaguaacua..................................................................................................   1   0   LT-3
    ............................................................................................................................................................................................................uuuugguaguuNgaucgugaaauu......................   1   1   JCK-1
    ............................................................................................................................................................................................................uuuugguaguuugaucgugaaauu......................   1   0   JCK-1
    .............................................................................................................................................................................................................uuugguaguuugaucgugGaauu......................   2   1   LCK-2
    ..............................................................................................................................................................................................................uugguaguuugaucgugGaauu......................   1   1   LCK-2
    ..............................................................................................................................................................................................................uugguaguuugaucgugGaauu......................   1   1   LT-1
    ...............................................................................................................................................................................................................ugguaguuugaucgugGaauu......................   1   1   JCK-3
    ................................................................................................................................................................................................................gguaguuugaucgugGaa........................   3   1   LT-1
    ................................................................................................................................................................................................................gguaguuugaucgugGaa........................   8   1   LCK-2
    ................................................................................................................................................................................................................gguaguuugaucgugGaau.......................   6   1   JCK-2
```

```
                                                                                                                                                       Star                                                        Mature
guaaaguagcuccugggugggaaacuacuccuuugaugggguaucgcaauggcccuuuuugcauuauuccuaucuauuauuuuggagauuuauacuucuuccauuuuacuggacaaauuuggaaugaauuagauucacaagaacuaguaacuagguuuucaauaaaaagugaagcauuuaggucuuugauuuuuaucccuuucuauuuugguaguuugaucgugaaauuucuuuguuuuguaguuuugaa
.................................................................................................................................................................................................................gguaguuugaucgugGaau......................   8      1   LT-3
.................................................................................................................................................................................................................gguaguuugaucgugGaau......................   14     1   JT-3
.................................................................................................................................................................................................................gguaguuugaucgugGaau......................   4      1   LCK-3
.................................................................................................................................................................................................................gguaguuugaucgugGaau......................   1      1   JCK-1
.................................................................................................................................................................................................................gguaguuugaucgugaaau......................   1      0   LT-2
.................................................................................................................................................................................................................gguaguuugaucgugGaau......................   18     1   LT-1
.................................................................................................................................................................................................................gguaguuugaucgugGaau......................   7      1   JT-2
.................................................................................................................................................................................................................gguaguuugaucgugUaau......................   7      1   JCK-2
.................................................................................................................................................................................................................gguaguuugaucgugUaau......................   2      1   JT-3
.................................................................................................................................................................................................................gguaguuugaucgugGaau......................   23     1   JT-1
.................................................................................................................................................................................................................gguaguuugaucgugUaau......................   3      1   JT-2
.................................................................................................................................................................................................................gguaguuugaucgugGaau......................   46     1   LCK-2
.................................................................................................................................................................................................................gguaguuugaucgugUaauu.....................   16     1   JT-3
.................................................................................................................................................................................................................gguCguuugaucgugaaauu.....................   1      1   JCK-1
.................................................................................................................................................................................................................gguaguuugaucgugaaauu.....................   18     0   JCK-2
.................................................................................................................................................................................................................gguaguuAgaucgugaaauu.....................   1      1   LT-3
.................................................................................................................................................................................................................gguaguuugaucgugaaauu.....................   1      0   LCK-2
.................................................................................................................................................................................................................gguaguuugaucgugaaauu.....................   9      0   JCK-3
.................................................................................................................................................................................................................gguaguuugaucgugaaauu.....................   4      0   LT-2
.................................................................................................................................................................................................................gguaguuugaucgugGaauu.....................   6      1   LT-2
.................................................................................................................................................................................................................gguaguuugaucgugUaauu.....................   6      1   JCK-2
.................................................................................................................................................................................................................gguaguuugaucgugGaauu.....................   1304   1   LCK-2
.................................................................................................................................................................................................................gguaguuugaucgugGaauu.....................   41     1   JT-2
.................................................................................................................................................................................................................gguaguuugaucgugGaauu.....................   7      1   LCK-1
.................................................................................................................................................................................................................gguaguuugaucgugGaauu.....................   199    1   LT-3
.................................................................................................................................................................................................................gguaguuugaucgugGaauu.....................   26     1   JCK-1
.................................................................................................................................................................................................................gguaguuugaucgugaaauu.....................   8      0   LCK-3
.................................................................................................................................................................................................................gguaguuAgaucgugaaauu.....................   1      1   JCK-1
.................................................................................................................................................................................................................gguaguuugaucgugGaauu.....................   8      1   JCK-3
.................................................................................................................................................................................................................gguaguuugaucgugGaauu.....................   203    1   LCK-3
.................................................................................................................................................................................................................gguaguuugaucgugGaauu.....................   120    1   JT-1
.................................................................................................................................................................................................................gguaguuugaucgugGaauu.....................   55     1   JCK-2
.................................................................................................................................................................................................................gguaguuugaucgugaaauu.....................   13     0   JCK-1
.................................................................................................................................................................................................................gguaguuGgaucgugaaauu.....................   1      1   LT-2
.................................................................................................................................................................................................................gguaguuugaucgugGaauu.....................   45     1   JT-3
.................................................................................................................................................................................................................gguaguuugaucgugUaauu.....................   1      1   LT-1
.................................................................................................................................................................................................................gguaguuugaucgugUaauu.....................   10     1   JT-2
.................................................................................................................................................................................................................gguaguuugaucgugUaauu.....................   5      1   JCK-1
.................................................................................................................................................................................................................gguaguuAgaucgugaaauu.....................   1      1   JT-3
.................................................................................................................................................................................................................gguaguuuAaucgugaaauu.....................   1      1   LT-1
.................................................................................................................................................................................................................gguaguuugaucgugaaauu.....................   3      0   LT-1
.................................................................................................................................................................................................................gguaguuugaucgugGaauu.....................   517    1   LT-1
.................................................................................................................................................................................................................gguaguuAgaucgugaaauu.....................   1      1   JCK-3
.................................................................................................................................................................................................................gguaguuugaucgugGaauuu....................   1      1   LT-3
.................................................................................................................................................................................................................gguaguuugaucgugGaauuu....................   1      1   LCK-3
.................................................................................................................................................................................................................gguaguuugaucgugaaauuu....................   1      0   JCK-2
.................................................................................................................................................................................................................gguaguuugaucgugaaauuu....................   1      0   LT-2
.................................................................................................................................................................................................................gguaguuugaucgugaaauuuc...................   1      0   JCK-2
.................................................................................................................................................................................................................gguaguuugaucgugaaauuucu..................   1      0   LT-1
.................................................................................................................................................................................................................gguaguuugaucgugaaauuucuu.................   1      0   LT-2
.................................................................................................................................................................................................................gguaguuugaucgugGaauuucuuu................   1      1   JCK-2
.................................................................................................................................................................................................................gguaguuugaucgugaaauuucuuu................   1      0   LT-3
.................................................................................................................................................................................................................gguaguuugaucgugaaauuucuuug...............   5      0   JCK-3
.................................................................................................................................................................................................................gguaguuugaucgugGaauuucuuug...............   2      1   LCK-2
.................................................................................................................................................................................................................Aguaguuugaucgugaaauuucuuug...............   1      1   LT-1
.................................................................................................................................................................................................................gguaguuugaucgugaaauuucuuug...............   1      0   JCK-2
.................................................................................................................................................................................................................gguaguuugaucgugaaauuucuuug...............   1      0   LT-1
.................................................................................................................................................................................................................gguaguuugaucgugaaauuucuuugu..............   2      0   JCK-1
.................................................................................................................................................................................................................gguaguuugaucgugaaauuucuuugu..............   1      0   JCK-2
.................................................................................................................................................................................................................ggGaguuugaucgugaaauuucuuugu..............   1      1   JCK-1
.................................................................................................................................................................................................................gguaguuAgaucgugaaauuucuuugu..............   2      1   JCK-2
.................................................................................................................................................................................................................gguaguuugaucgugaaauuucuuugu..............   2      0   LT-2
.................................................................................................................................................................................................................gguaguuugaucgugGaauuucuuuguu.............   1      1   LCK-2
.................................................................................................................................................................................................................gguaguuugaucgugaaauuucuuuguu.............   1      0   JCK-3
.................................................................................................................................................................................................................gguaguuuAaucgugaaauuucuuuguu.............   1      1   LT-3
.................................................................................................................................................................................................................gguaguuugaucgugGaauuucuuuguu.............   1      1   JCK-3
.................................................................................................................................................................................................................gguaguuugaucgugaaauuucuuuguu.............   1      0   LT-1
.................................................................................................................................................................................................................gguaguuAgaucgugaaauuucuuuguuu............   1      1   LT-1
.................................................................................................................................................................................................................gguaguuAgaucgugaaauuucuuuguuu............   1      1   JCK-2
.................................................................................................................................................................................................................gguaguuugaucgugaaauuucuuuguuu............   3      0   JCK-2
```

```
                                                                                                                                                      Star                                                                      Mature
guaaaguagcuccugggugggaaacuacuccuuugauggguaucgcaauggcccuuuuugcauuauuccuaucuauuauuuuggagauuuauacuucuuccauuuuacuggacaaauuuggaaugaauuagauucacaagaacuaguaacuagguuuucaauaaaaagugaagcauuuaggucuuugauuuuuaucccuuucuauuuugguaguuugaucgugaaauuucuuuguuuuuguaguuuugaa
................................................................................................................................................................................................................gguaguuugaucgugGaauuucuuuguuu.............   1   1   LT-1
................................................................................................................................................................................................................gguaguuugaucgugaaauuucuuuguuu.............   1   0   JCK-3
................................................................................................................................................................................................................gguaguuugaucgugaaauuucuuuguuu.............   1   0   JCK-1
................................................................................................................................................................................................................gguaguuAgaucgugaaauuucuuuguuu.............   1   1   LT-3
................................................................................................................................................................................................................gguaguuCgaucgugaaauuucuuuguuuu............   1   1   LT-2
................................................................................................................................................................................................................gguaguuCgaucgugaaauuucuuuguuuu............   3   1   JCK-2
................................................................................................................................................................................................................gguaguuCgaucgugaaauuucuuuguuuu............   1   1   JCK-1
................................................................................................................................................................................................................gguaguuCgaucgugaaauuucuuuguuuu............   1   1   JCK-3
.................................................................................................................................................................................................................guaguuugaucgugGaauu......................   1   1   LT-1
........................................................................................................................................................................................................................gaucgugaaauuucuuug................   1   0   LT-3
........................................................................................................................................................................................................................gaucgugaaauuucuuuguuu.............   1   0   JCK-2
........................................................................................................................................................................................................................gaucgugaaauuucuuuguuu.............   4   0   JCK-3
.........................................................................................................................................................................................................................aucgugaaauuucuuugu...............   2   0   LT-2
.........................................................................................................................................................................................................................aucgugaaauuucuuuguu..............   3   0   LT-3
.........................................................................................................................................................................................................................aucgugaaauuucuuuguuu.............   1   0   LT-2
.........................................................................................................................................................................................................................aucgugaaauuucuuuguuu.............   1   0   LT-3
.........................................................................................................................................................................................................................aucgugaaauuucuuuguuu.............   2   0   LT-1
.........................................................................................................................................................................................................................aucgugaaauuucuuuguuu.............   3   0   JCK-1
..........................................................................................................................................................................................................................ucgugaaauuucuuuguu..............   1   0   JCK-2
..........................................................................................................................................................................................................................ucgugaaauuucuuuguu..............   5   0   LCK-3
...........................................................................................................................................................................................................................cgugaaauuucuuuguuu.............   2   0   LT-2
.................................................................................................................................................................................................................................auuucuuuguuuCuguaguuu....   1   1   LT-3
```
